# The art of tartness: the genetics of organic acid content in fresh fruits

**DOI:** 10.1093/hr/uhae225

**Published:** 2024-08-06

**Authors:** Shixue Miao, Xiaoyu Wei, Lingcheng Zhu, Baiquan Ma, Mingjun Li

**Affiliations:** State Key Laboratory for Crop Stress Resistance and High-Efficiency Production/Shaanxi Key Laboratory of Apple, College of Horticulture, Northwest A&F University, Yangling 712100, Shaanxi, China; State Key Laboratory for Crop Stress Resistance and High-Efficiency Production/Shaanxi Key Laboratory of Apple, College of Horticulture, Northwest A&F University, Yangling 712100, Shaanxi, China; State Key Laboratory for Crop Stress Resistance and High-Efficiency Production/Shaanxi Key Laboratory of Apple, College of Horticulture, Northwest A&F University, Yangling 712100, Shaanxi, China; State Key Laboratory for Crop Stress Resistance and High-Efficiency Production/Shaanxi Key Laboratory of Apple, College of Horticulture, Northwest A&F University, Yangling 712100, Shaanxi, China; State Key Laboratory for Crop Stress Resistance and High-Efficiency Production/Shaanxi Key Laboratory of Apple, College of Horticulture, Northwest A&F University, Yangling 712100, Shaanxi, China

## Abstract

Organic acids are major determinants of fruit flavor and a primary focus of fruit crop breeding. The accumulation of organic acids is determined by their synthesis, degradation, and transport, all of which are manipulated by sophisticated genetic mechanisms. Constant exploration of the genetic basis of organic acid accumulation, especially through linkage analysis, association analysis, and evolutionary analysis, have identified numerous loci in recent decades. In this review, the genetic loci and genes responsible for malate and citrate contents in fruits are discussed from the genetic perspective. Technologies such as gene transformation and genome editing as well as efficient breeding using marker-assisted selection (MAS) and genomic selection (GS) are expected to break the bottleneck of traditional fruit crop breeding and promote fruit quality improvement.

## Introduction

Fresh fruits make up a large proportion of a healthful human diet. Fruit flavor is a primary factor affecting consumption behavior and has been a major focus of genetic research and breeding practices [1–3]. Sugars and organic acids determine the sweet–sour taste of fruit and thus work together to influence fruit flavor; however, sweet–sour taste perception is more affected by organic acids according to sensory evaluations [4–7]. Therefore, organic acids play a vital role in flavor improvement of fruit crops [8]. Mechanisms and regulation of organic acid accumulation in fruit crops have been well reported and reviewed [9, 10]. Nevertheless, it remains uncertain how biochemical pathway-related genes and regulation factors involved in organic acid accumulation can be applied to breeding practices. Forward genetics promotes the understanding of the genetic basis of phenotypic traits, as well as reveals natural genetic variations, which can be utilized in marker-assisted selection (MAS) to support traditional breeding and in genome engineering. Understanding of the genetics of organic acid content will aid in developing more efficient strategies for fruit crop breeding and provide more promising approaches for fruit flavor improvement.

As an important indicator of fruit flavor, the content of organic acids has been a main selection target during fruit crop domestication and improvement. Domestication syndrome traits associated with organic acid content have been broadly reported in fruit crops such as apple (*Malus domestica* Borkh.) [11], peach (*Prunus persica* L.) [2], mandarin (*Citrus reticulata* Blanco) [12], Chinese cherry (*Prunus pseudocerasus* (Lindl.) G.Don) [13], and tomato (*Solanum lycopersicum* L.) [1]. Organic acid content is a classic quantitative trait controlled by multiple genes. Quantitative trait locus (QTL) mapping, genome-wide association studies (GWAS), and evolutionary analysis in natural populations have been widely employed to explore genetic loci and variations associated with fruit organic acid content [8, 14–20]. Although endeavors in exploring the genetic basis underlying organic acids have been consistently reported, there are few available overviews on the genetic loci, genes, and corresponding mechanisms related to organic acid accumulation. In this review, progress in understanding the organic acid content of fruit crops is summarized from a genetic perspective with the hope of more efficient and precise breeding for fruit quality improvement in the near future.

## Metabolism and transport of organic acids in fruits

**Figure 1:**
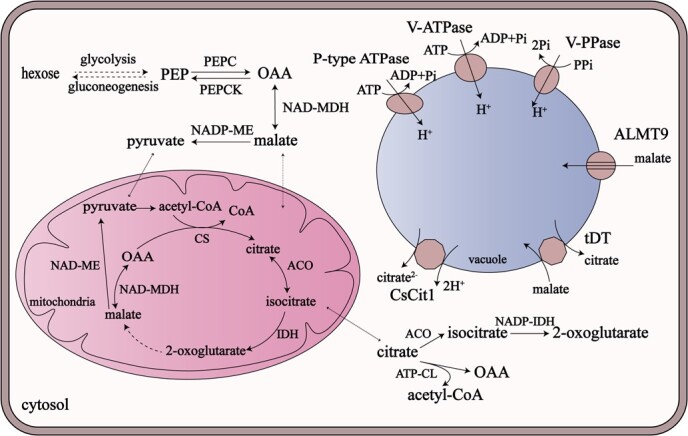
Metabolism and transport of malate and citrate in fruit mesocarp cells. Only the metabolites, enzymes, and transporters mentioned in this review are shown. Enzymes involved in metabolism: ACO, aconitase; ATP-CL, ATP-citrate lyase; CS, citrate synthase; IDH, isocitrate dehydrogenase; NADP-IDH, NADP-dependent isocitrate dehydrogenase; NAD-MDH, NAD-dependent malate dehydrogenase; NAD-ME, NAD-dependent malic enzyme; NADP-ME, NADP-dependent malic enzyme; OAA, oxaloacetate; PEP, phosphoenolpyruvate; PEPC, phosphoenolpyruvate carboxylase; PEPCK, phosphoenolpyruvate carboxykinase. Vacuolar transporters: ALMT9, aluminum-activated malate transporter 9; CsCit1, *Citrus sinensis* citrate transporter 1; tDT, tonoplast dicarboxylate transporter; V-ATPase, vacuolar-type H^+^-ATPase; V-PPase, vacuolar-type H^+^-PPase.

The main organic acids in fruits are malic acid and citric acid. The predominant type of organic acid varies among species. For instance, malic acid is dominant in apple [21], peach [22], loquat (*Eriobotrya japonica* Lindl.) [23], and sweet cherry (*Prunus avium* L.) [24], while citric acid is the most abundant acid in citrus (*Citrus* spp.) [25]. Malate and citrate are technically defined as the conjugated base of malic acid and citric acid respectively; however, these two terms are generally used to describe all physiological forms of the two acids [10, 23, 26]. The consensus is that the accumulation of malate and citrate in fruit is determined by synthesis, degradation, and transport (Fig. 1).

Biosynthesis of malate relies on two key enzymes, cytosolic NAD-dependent malate dehydrogenase (NAD-cytMDH) and phosphoenolpyruvate carboxylase (PEPC). The glycolysis product phosphoenolpyruvate (PEP) is carboxylated by PEPC into oxaloacetate (OAA), and OAA can then be reduced by NAD-cytMDH to malate in the cytosol [27–30]. Malate degradation occurs in two ways: a reverse of its synthesis or direct decarboxylation. The first degradation route is initiated by the oxidation of malate into OAA by NAD-cytMDH, followed by decarboxylation of OAA by phosphoenolpyruvate carboxykinase (PEPCK), resulting in the production of PEP, which is associated with gluconeogenesis. The second way that malate is degraded is direct decarboxylation by cytosolic NADP-dependent malic enzyme (NADP-cytME) into pyruvate, which can also be converted into PEP or transported into the mitochondria [31–33].

Malate produced in the cytosol can be transported into mitochondria to participate in the tricarboxylic acid (TCA) cycle, where conversions between malate and citrate take place. Mitochondrial citrate synthase (mtCS) directly controls citrate synthesis, while mitochondrial aconitase (mtACO) and mitochondrial NAD-dependent isocitrate dehydrogenase (NAD-mtIDH) are crucial to citrate degradation in the TCA cycle [10, 34]. Citrate synthesized by the TCA cycle can be degraded in the cytosol through the γ-aminobutyrate (GABA) synthesis pathway and acetyl-CoA catabolism. Two key enzymes of the GABA shunt are cytosolic aconitase (cytACO), which catalyzes the conversion of citrate into isocitrate, and cytosolic NADP-dependent isocitrate dehydrogenase (NADP-cytIDH), catalyzing the conversion of isocitrate into 2-oxoglutarate. The key enzyme of the acetyl-CoA catabolism pathway is ATP-citrate lyase (ATP-CL), which catalyzes the conversion of citrate into OAA and acetyl-CoA [10, 35].

In the flesh of fruits, the vacuole of each cell occupies up to 90% of the cell volume, and the majority of sugars and acids are transported across the tonoplast and stored in the vacuole under sophisticated regulation [10, 36, 37]. Therefore, transport of organic acids is crucial for their accumulation in fruits [38]. Tonoplast transporters – channels, carriers, and proton pumps – dominate the process of organic acid transport. Aluminum-activated malate transporter 9 (ALMT9) has been reported to be a malate channel involved in malate transport from the cytosol into the vacuole in tomato [39], apple [40, 41], grape (*Vitis vinifera* L.) [42], and pear (*Pyrus* spp.) [43]. It has also been found that *Sl-ALMT9* is associated with citrate content in tomato [1] and that ALMTs are responsible for citrate content in kiwifruit (*Actinidia* spp.) [44] and citrus [45]. The tonoplast dicarboxylate transporter (tDT) was first identified as a malate carrier in *Arabidopsis* [46]. In apple, MdtDT plays a role in malate transport into the vacuole [47]. Moreover, a genetic study on the flavor of apple fruit revealed that *MdtDT* could downregulate citrate accumulation and that the selection of *MdtDT* during domestication causes a dramatic decrease in the citrate content of apple fruit [19]. In citrus, citrate transporter 1 (CsCit1), a homolog of AttDT, was identified as a citrate^2−^/H^+^ symporter mediating the efflux of citrate^2−^ from the vacuole [48]. In addition to channels and carriers like those mentioned above, proton pumps also play vital roles in organic acid accumulation.

In the cytosol, almost all malate is in the dianion form and most citrate in the trianion form. Once malate and citrate are transported into the acidic vacuole, they are immediately protonated. This maintains the electrochemical potential gradients of malate and citrate across the tonoplast, thereby permitting their continuous transport into the vacuole [10]. The efficiency of this ‘acid trap’ mechanism relies on the acidic vacuolar pH and the potential gradient across the tonoplast, both of which are produced by the transport of protons into the vacuole via proton pumps. Three types of tonoplast proton pumps are involved in organic acid accumulation, namely V-ATPase, V-PPase, and P-type ATPase [9].

**Table 1:** Genetic studies and candidate genes for malate content in apple.

Chr	QTL	Population	Candidate gene(s)	Gene function	Variation and marker	Reference
16	*Ma*	‘Prima’ × ‘Fiesta’				[49]
16	*Ma*	‘Fiesta’ × ‘Discovery’				[8]
16	*Ma*	‘Telamon’ × ‘Braeburn’				[50]
16	*Ma*	‘Royal Gala’ × ‘PI 613988’ ‘Royal Gala’ × ‘PI 613971’				[14]
16	*Ma*	‘Royal Gala’ × ‘PI 613978’ ‘Royal Gala’ × ‘PI 613979’ ‘Royal Gala’ × ‘PI 613971’ ‘Royal Gala’ × ‘PI 613988’	*Ma1*, *Ma2*	ALMT9, malate transporter	A/G in the CDS (nonsense mutation), CAPS_1455_	[40]
16	*Ma*	‘Prima’ × ‘Fiesta’	*Ma1*			[51]
16	*Ma*	‘Zisai Pearl’ × ‘Red Fuji’	*MdALMTII* (*Ma1*)			[16]
16	*Ma*	‘Jiguan’ × ‘Wangshanhong’				[15]
16	*Ma*	16 *F*_1_ full-sib families			ss475882553 (GDsnp01588)	[52]
16	*Ma*	‘Nicoter’ × ‘Cripps Pink’ ‘Zari’ × ‘Fuji’ ‘B3F44’ × ‘B3FS1’				[53]
16	*Ma*	353 apple accessions	*MdMYB21*	MYB TF, downregulates *Ma1*	2-bp InDel in the promoter WBG82	[54]
8	*Ma3*	‘Fiesta’ × ‘Discovery’				[8]
8	*Ma3*	‘Telamon’ × ‘Braeburn’				[50]
8	*Ma3*	‘Jonathan’ × ‘Golden Delicious’				[55]
8	*Ma3*	‘Jonathan’ × ‘Golden Delicious’	*MDP0000582174* ^a^ (*MdMYB4*), *MDP0000239624*^a^ (*MdME*)	MYB TF, malic enzyme		[56]
8	*Ma3*	‘Jiguan’ × ‘Wangshanhong’				[15]
8	*Ma3*	‘Jonathan’ × ‘Golden Delicious’	*MdPP2CH* *MdSAUR37*	Inactivates tonoplast V-ATPases and Ma1 via dephosphorylation Suppresses dephosphotase activity of MdPP2CH	A/G SNP in the CDS A 184-bp InDel marker 36-bp InDel in the promoter	[16]
8	*Ma3*	‘Jonathan’ × ‘Golden Delicious’	*MdMYB44*	MYB TF, represses *Ma1*, *Ma10*, *MdVHA*	A/G and T/− in the promoter	[16, 57]
8	*Ma3*	16 *F*_1_ full-sib families				[52]
8	*Ma3*	‘Nicoter’ × ‘Cripps Pink’ ‘Zari’ × ‘Fuji’ ‘B3F44’ × ‘B3FS1’			AX-105220211	[53]
6	*Ma4*	‘Royal Gala’ × ‘PI 613988’			HRM-Ma4/SSR-Ma4	[58]
6	*Ma4*	‘Nicoter’ × ‘Cripps Pink’ ‘Zari’ × ‘Fuji’ ‘B3F44’ × ‘B3FS1’				[53]
1	*Ma5*	‘Nicoter’ × ‘Cripps Pink’ ‘Zari’ × ‘Fuji’ ‘B3F44’ × ‘B3FS1’				[53]

(Continued)

**Table 1:** Continued

Chr	QTL	Population	Candidate gene(s)	Gene function	Variation and marker	Reference
4	*Ma6*	‘Royal Gala’ × ‘PI 613988’			HRM-Ma6/SSR-Ma6	[58]
13	*Ma7*	‘Honeycrisp’ × ‘Qinguan’	*MDH1*	Malate dehydrogenase, upregulated by *MdbHLH74*	144-bp InDel in the promoter, contains an E-box binding motif of *bHLH74*	[59]
15		‘Gala’ × ‘Xiahongrou’	*MdNADP-ME*	Malic enzyme, repressed by *MdMYB2*	A/C SNP in the promoter, alters the binding of *MdMYB2* to the promoter	[60]
2, 10, 13, 15, 17		‘Telamon’ × ‘Braeburn’				[50]
1		‘Royal Gala’ × ‘PI 613988’				[14]
16		461 *Malus* accessions	*Ma1*			[19]
15		270 *Malus* accessions	*PH4* ^a^	MYB TF, activates vacuolar acidification		[3]
2		344 *Malus* accessions				[61]
2, 14, 15, 16		117 *Malus* accessions	*MDH* ^a^, two *ALMTs*^a^, *Ma1*			[62]
16		91 *Malus* accessions	*Ma1*			[63]
17			*Ma10*	Tonoplast P_3A_-ATPase, facilitates malate transport	A/T and A/G in the promoter	[64]
2			*MdMYB123*	MYB TF, upregulates *Ma1* and *MdMa11* (a P_3A_-ATPase)	CDS: A/T (nonsense mutation)	[65]

a Candidate genes that were proposed but not verified.

## Genetic case studies of organic acid content in fruits

### A representative of fruits high in malate: apple

Apple is a fruit rich in malate and has been subject to a considerable number of genetic studies of malate content (Table 1). Major QTLs associated with apple fruit acidity or malate content are on linkage group (LG) 16 [8, 14–16, 49, 50, 53] and LG 8 [8, 15, 16, 50, 53, 55, 56]. Maliepaard *et al*. [49] constructed linkage maps of the apple cultivars ‘Prima’ and ‘Fiesta’ and first mapped the malate locus *Ma* to the distal end of LG 16. The availability of the draft sequence of the apple genome [66] allowed the development of sequence-based markers used for fine mapping. Afterwards, locus *Ma* was narrowed down to a region of <150 kb in the ‘Golden Delicious’ genome utilizing new simple sequence repeat (SSR) markers [14]. *Ma1*, encoding an aluminum-activated malate transporter, was subsequently identified as a major gene at the *Ma* locus controlling apple fruit acidity [40, 51]. Furthermore, a single-nucleotide mutation (G to A) at base 1455 in the *Ma1* coding sequence, which leads to the presence of a premature stop codon, was found to be highly correlated with the low-acidity trait in apple [40]. Ma1, also MdALMT9, is localized to the tonoplast and participates in malate transport into the vacuole. Its truncated protein, ma1, has significantly lower transport activity than Ma1 because of the lack of a conserved C-terminal domain [41]. Intriguingly, the latest study found that the overexpression of *Ma1* caused a dramatic decline in malate content of apple fruits, resulting in the discovery of alternative splicing in *Ma1* and mechanisms of two isoforms, Ma1α and Ma1β, functioning in malate transport [67].

Progress in next-generation sequencing and the availability of the apple reference genome facilitated population genetic analysis and genome-wide association studies (GWAS) of large natural populations, providing enormous opportunities for exploring genetic variations and identifying candidate genes underlying important traits. Research in this area suggested that *Ma1* has undergone selection during apple domestication [19, 62, 63]. In addition to *Ma1*, two *ALMT* genes and an *MDH* gene were identified in selection sweeps based on genome re-sequencing of 117 *Malus* accessions [62]. Recently, the MYB-type transcription factor gene *MdMYB21* was identified in the *Ma* locus by region-based association mapping. MdMYB21 negatively regulates malate content by repressing the expression of *Ma1*, and a 2-bp insertion/deletion (InDel) in the *MdMYB21* promoter altered its expression level and regulation of malate content [54]*.*

In addition to the *Ma* locus on chromosome 16, another major QTL for apple malate content has been identified on chromosome 8, which was named *Ma3* following two ALMT-like genes *Ma1* and *Ma2* under the *Ma* locus [40, 52]. In a segregating population from the cross between the apple cultivars ‘Fiesta’ and ‘Discovery,’ fruit acidity was almost completely explained by two QTLs on LGs 8 and 16 [8]. In addition, a QTL for malic acid was also detected on LG 8 of ‘Jonathan’ by a study using an SSR linkage map of a population resulting from a ‘Jonathan’ × ‘Golden Delicious’ cross [55]. Following this, a dense SNP genetic map of the same population was constructed and *MdMYB4* and *MdME* in a QTL on LG 8 were proposed to be correlated with fruit acidity [56]. However, no further gene function research was done for these two candidate genes in relation to malate content. Furthermore, a study combined with QTL mapping and bulk segregant analysis using next-generation sequencing (BSA-seq) identified four major QTLs (qtl08.1, qtl08.2, qtl08.3, qtl16.1) for malate content. In addition to *MdALMT9* in qtl16.1, *MdSAUR3*, *MdPP2CH* and *MdMYB44* were identified in QTLs on LG 8, and malate-related allelic variations of these three genes were verified [16, 57]. Subsequently, *Ma3* was also reported by Verma *et al*. [52] and Rymenants *et al*. [53], with no candidate genes proposed.

In addition to *Ma* (locus on chromosome 16) and *Ma3* (locus on chromosome 8), other QTLs controlling apple fruit acidity have been identified. Two QTLs associated with high fruit acidity, localized on chromosomes 4 (*Ma6*) and 6 (*Ma4*), were identified using pooled genome sequencing analysis and an allele frequency directional difference and density (AFDDD) mapping approach [58]. However, no candidate genes under these two loci were suggested. In addition, a QTL on LG 1 contributing to apple fruit acidity, named *Ma5*, was reported [53]. In a recent study, *Ma7* on chromosome 13 was detected as a major locus controlling apple malate content using a hybrid population from two apple cultivars, ‘Honeycrisp’ and ‘Qinguan’, with the same *Ma1* genotype (*Ma1*/*Ma1*), and a 144-bp InDel in the promoter of the candidate gene, *MDH1*, was reported to be responsible for differences in malate content of apple cultivars [59]. In addition, *MdNADP-ME* in a stable QTL on chromosome 15 was identified and an A/C SNP affecting the binding ability of *MdMYB2* to its promoter was verified to be related to malate content [60]. Some minor loci controlling apple acidity were also detected on LGs 2, 10, 13, 15, and 17 [50] and LGs 1 and 6 [14].

An association signal for malate was found on chromosome 2 in a recent GWAS study [61]. More recently, a metabolome-based genome-wide association study (mGWAS) utilizing 270 wild and cultivated apple accessions showed that a vacuole acidification-related gene, *PH4*, was located on chromosome 15 in a region under selection during domestication [3]. Moreover, comparative transcriptome analysis and functional studies revealed that natural mutations in *Ma10* and *MdMYB123* were partially responsible for observed malate content differences in apple germplasm [64, 65]. Ma10, a P_3A_-ATPase located in the tonoplast, was determined to facilitate the transport of malate into the vacuole by cooperating with Ma1; two SNPs (A/T and A/G) in the promoter of *Ma10* were found to be associated with malic acid content [64]. Furthermore, a nonsense mutation (A to T) was found in the coding sequence of *MdMYB123* and the truncated protein of MdMYB123 was reported to compete with normal protein for binding to the promoter of *Ma1* and *MdMa11* (a P_3A_-ATPase gene), resulting in decreased malate accumulation [65]*.*

### A representative of fruits high in citrate: citrus

Citric acid accounts for more than 90% of total titratable acids in citrus juice [68]. In citrus fruits, vacuolar P-ATPases (CitPH1 and CitPH5) and transcription factors CitAN1 (also Noemi, in the bHLH family), CitPH3 (a WRKY), CitPH4 (an R2R3-MYB), and CitTRL (an R3-MYB) are proposed to be responsible for the variance in acidity [69, 70]. *CitPH1* and *CitPH5* were expressed in sour varieties of lemon (*Citrus limon* (L.) Burm.f.), orange (*C. sinensis* (L.) Osbeck), pummelo (*C. maxima* (Burm.) Merr*.*), and rangpur lime (*C. limonia* Osbeck) fruits, while their expression levels were dramatically low in sweet-tasting varieties where citric acid content was reduced [69]. Earlier work in petunia (*Petunia hybrida* Vilm.) provides more clues about acidity regulation of citrus. In petunia, seven *PH* loci (*PH1*–*PH7*) control vacuolar pH or acidity. PH5 is a P_3A_-ATPase residing in the tonoplast, and *PH1* encodes a P_3B_-ATPase that has no known independent transport activity but physically interacts with PH5 and promotes its proton-pump activity [71, 72]. PH3, PH4, and AN1 are the upstream transcription factors of *PH1* and *PH5* [71, 73].

Genetic studies on citrate content QTLs are relatively limited in citrus (Table 2). *CitAN1* (*Noemi*) was demonstrated to be a major determinant of citrus fruit acidity by genetic analysis of accessions of citron (*Citrus medica* L.), limetta (*C. limetta* Risso), sweet lime (*C. limettioides* Tan.), lemon, and sweet orange. Large deletions or insertions of retrotransposons in *CitAN1* were responsible for the ‘acidless’ phenotype, which is characterized by an extreme decrease in fruit acidity [74]. Likewise, it was found that mutations in *CitAN1* led to the reduced expression of *CitPH1* and *CitPH5* and thus low acidity in the sweet fruits of ‘Faris’ and other ‘acidless’ citrus fruits [69]. However, Huang *et al*. [75] reported that *CitPH4*, but not *CitAN1*, played a vital role in the accumulation of citric acid in citrus fruits. *CitPH4* was highly correlated with citric acid levels when analyzing genomic variation data from the pangenome and fruit transcriptome data from *Citrus* and *Citrus*-related genera, and the overexpression of *CitAN1* was unable to enhance the citric acid level in citrus calli with low expression of *CitPH5* [75]. Despite the central roles of two ATPases (PH1 and PH5) in citrate content regulation of citrus, there have been no natural variations discovered in these two genes to date. Furthermore, the exact mechanisms by which PH1 and PH5 drive the transport of citrate into the vacuole remain unclear and the discovery of citrate transporters cooperating with these two ATPases is still an urgent issue.

**Table 2:** Genetic studies and candidate genes for citrate content in citrus.

Material	Candidate gene(s)	Gene function	Variation and marker	Reference
Lemon and orange	*CitAN1*	bHLH TF, upregulates *CitPH1* and *CitPH5*	Large deletions and/or transposon insertions	[69]
Citron, limetta, sweet lime, and sweet orange	*CitAN1*	bHLH TF, upregulates *CitPH1* and *CitPH5*	Large deletions or transposon insertions	[74]
314 citrus accessions	*PH4*	MYB TF, upregulates *CitPH1* and *CitPH5*	Promoter: insertions; CDS: SNPs	[75]
Mandarin	*ACO* ^a^	Key enzyme in citrate degradation		[12]
Mandarin, pummelo	*NAD-mtIDH* ^a^	Key enzyme in citrate degradation		[76]
111 citrus accessions and 35 full-sib families with 676 individuals	*Glutamate dehydrogenase* ^a^	Catalyzes the reversible conversion of glutamate to α-ketoglutarate		[77]
*F*_1_ population from a cross between a clementine and a mandarin cultivar	*Phosphofructokinase* ^a^	Rate-limiting enzyme in glycolysis	Marker: PKF-M-186	[78]

a Candidate genes that were proposed but not verified.

Furthermore, genes involved in the TCA cycle are closely related to citric acid accumulation in citrus [79]. A study on mandarin oranges indicated that *ACO* alleles were under selection during its domestication process [12]. Another study on the origin and evolution of citrus demonstrated that a major locus at the start of chromosome 8 was associated with palatability of mandarin and that a *mitochondrial isocitrate dehydrogenase* (*NAD-mtIDH*) was detected among the candidate genes [76]. In addition, *glutamate dehydrogenase* and *phosphofructokinase* genes were potentially responsible for citric acid content according to GWAS of combined parental and breeding populations and QTL mapping of a segregating population from a cross between clementine (*Citrus clementina* Hort. ex Tan.) and mandarin, respectively [77, 78]. Other QTLs associated with acidity were reported through linkage analysis of citrus hybrid populations, but no additional candidate genes were proposed [80–82].

### A model fruit: tomato

Tomato is widely used as a model crop for the study of fruit development and ripening [83]. Malic acid and citric acid, constituting over 90% of the total organic acids, largely determine the acidic taste of tomato fruit [83, 84]. GWAS on malate and citrate content of tomato fruits have been broadly reported (Supplementary Data Table S1). A significant SNP associated with malate content was located on chromosome 6 [1, 39, 85–88] and was designated *TFM6* (*TOMATO FRUIT MALATE ON CHROMOSOME 6*) [39]. *Sl-ALMT9* was identified as the key gene in the *TFM6* locus responsible for the variation in malate accumulation of tomato fruit, and a 3-bp InDel in a W-box motif within the promoter of *Sl-ALMT9* showed significant correlation with malate content. This 3-bp deletion can interrupt the binding of the WRKY transcriptional repressor Sl-WRKY42 to the *Sl-ALMT9* promoter, thereby weakening the repression of *Sl-ALMT9* expression and facilitating the accumulation of malate in tomato fruits [39]. In the proximity of *Sl-ALMT9* (*Solyc06g072920*), a polymorphism in or close to *Solyc06g072930.2* may be associated with malate content [87]. In addition, an InDel upstream of *Solyc06g072840*, encoding hydrogen peroxide-induced protein 1, was also associated with malate content [89]. A multiple-model GWAS has identified 15 genetic loci and candidate genes associated with malate content [88]. Furthermore, meta-analysis of GWAS revealed that *GTF* (*Glycosyl transferase group 1*) on chromosome 2 and *Sl-ALMT9* on chromosome 6 were associated with both malate and citrate content in tomato fruits [1]. Citrate, another major organic acid in tomato fruit, has a greater impact on consumer preferences [1]. Recently, 11 genetic loci and candidate genes associated with citrate content were identified in a GWAS based on SNPs, InDels, and structural variants (SVs) [90]. In tomato association mapping, almost every chromosome was detected to contain association loci for malate, citrate, total organic acids, or pH [1, 88, 91, 92]. Nevertheless, no other reliable candidate genes were identified.

Extensive QTL mapping for fruit flavor traits has been conducted using different biparental populations in tomato [93–99]. QTLs responsible for malic acid and citric acid content of tomato fruit were identified on most chromosomes (Supplementary Data Table S2). For malic acid, *ma1.2*, *ma1.3*, *ma2.1*, and *ma7.1* were consistently detected in more than one population, suggesting there were major genetic variations associated with malic acid content within these loci. The QTLs *cca2.1*, *ca-5E*, *cca6.3*, *cca7.1*, *cca8.1*, *ca-9F*, and *ca*-*10B* may include major determinants of citric acid content. It is worth noting that the loci *ma1.1*, *ma3.2*, *ma3.3*, and *cca3.3* overlapped those found by Tieman *et al*. [86] in heirloom tomatoes. Furthermore, QTLs controlling malic acid and citric acid were co-localized on chromosomes 1, 3, 6, and 7.

### Other fruits

As discussed earlier, *ALMT9* plays a key role in malate accumulation in apple and tomato fruits. In genetic studies of other fruits (Table 3), *ALMT*s were also reported. Population genomic analyses of 564 peach accessions demonstrated that *PpALMT1* (*Pp.LH.06G01819*) was located in a selective sweep region and further expression analysis combined with transient expression assays showed that *PpALMT1* positively regulated malate content in peach fruits [101]. In addition, *ZjALMT4* was reported to be responsible for the difference in malate content between jujube (*Ziziphus jujuba* Mill.) and sour jujube (*Z. jujuba* var. *spinosa* Hu); gene function analysis revealed that an SNP in the promoter of *ZjALMT4* altered the expression level of *ZjALMT4*, resulting in different malate contents between sour jujube and jujube [108]. In grape, two *ALMT*s were also detected in a QTL for malate content on chromosome 6 [104]. In addition to ALMTs, two types of MDH were also reported in grape. Reshef *et al*. [102] successfully detected two stable QTLs for malate content (on chromosomes 7 and 17) and discovered a *mitochondrial MDH* gene in the QTL on chromosome 17, which had also been suggested as a candidate gene by Bayo-Canha *et al*. [103]. Besides, two *cytoplasmic MDH* genes were proposed in a QTL on chromosome 1 for malate content in grape berries [104].

**Table 3:** Genetic loci and candidate genes for organic acids in peach, grape, pear, sweet melon, and jujube.

Species	Locus	Trait	Candidate genes	Gene function	Variation and marker	Reference
Peach	*D*	Malate and total acid	*ppa006339m* ^a^ (*Prupe*.*5G004300*)	Auxin efflux carrier		[17]
Peach	*D*	Malate and citrate	*PpRPH* (*Prupe.5G008400*)	A small protein	CDS: G/A SNP (nonsense mutation)	[100]
Peach		Malate	*PpALMT1* ^a^ (*Pp.LH.06G01819*)	Malate transporter		[101]
Peach		Malate, citrate, and quinate	*PpTST1* (*Prupe.5G006300*)	Tonoplast sugar transporter	G/T SNP in the CDS Marker: dCAPS1584	[20]
Grape		Malate	*MDH* ^a^ (*VIT_00007997001*)	Mitochondrial malate dehydrogenase		[102, 103]
Grape		Malate	Two *cytMDHs*^a^ (*Vitvi01g02239*, *Vitvi01g02240*)	Cytoplasmic malate dehydrogenase		[104]
Grape		Malate	Two ALMTs^a^ (*Vitvi06g00922*, *Vitvi06g00928*)	Malate transporter		[104]
Grape		Tartrate	*VIT_00010644001* ^a^	l-Idonate 5-dehydrogenase, key enzyme in tartrate synthesis		[103]
Asian pear		Citrate	*Pbr014969.1* ^a^	A homolog of the ATP-CL, key enzyme in citrate degradation		[105]
European pear		Malate	*Pbr013232.1* ^a^, *Pbr013272.1*^a^, and *Pbr030186.1*^a^			[105]
Sweet melon *Cucumis melo*		Malate and citrate	*CmPH*	PIN H^+^/auxin transporter	12-bp InDel in the CDS (a four amino-acid duplication)	[106]
*Cucumis melo* ssp. *melo*		Acidic/non-acidic	*CmPH* (*MELO3C025264*)	PIN H^+^/auxin transporter	12-bp InDel in the CDS (a four amino acid duplication)	[107]
*C. melo* spp. *agrestis*	–	Acidic/non-acidic	*MELO3C011482* ^a^	ATP-CL, key enzyme in citrate degradation		[107]
Jujube		Malate	*ZjALMT4*	Malate transporter	(C/G)/A SNP in the promoter, alters the binding of ZjWRKY7 to the promoter	[108]
Jujube		Citrate	*ZjACO3*	Cytoplasmic aconitase, participates in citrate degradation	G/A SNP in the promoter, alters the binding of ZjbHLH113 to the promoter	[109]

a Candidate genes that were proposed but not verified.

The consistency of reports about ALMTs in fruit crops reveals that malate accumulation is largely determined by its transport from the cytosol to the vacuole. In contrast, it seems that citrate metabolism, especially degradation, plays the vital role in the citrate accumulation of fruit crops since more metabolism-related genes were reported in genetic studies on citrate content. An SNP (G/A) in the promoter of *ZjACO3* was found to be responsible for the difference in citrate content between jujube and sour jujube [109]. In addition, *Pbr014969.1*, a homolog of *ATP-CL*, was identified in selection sweeps of Asian pears, while three malate-related genes were identified in European pears [105]. In sweet melon (*Cucumis melo* L.), citrate is the major acid [110]. A four amino-acid duplication in *CmPH*, encoding a PIN H^+^/auxin transporter, was found to be associated with the non-acidic phenotype of melon accessions [106]. Furthermore, it was reported that *CmPH* contributed to the acidity in the cultivated *melo* group (*C. melo* ssp. *melo*), while the acidity of the cultivated *agrestis* group (*C. melo* ssp. *agrestis*) might be attributed to the *ATP-CL* gene *MELO3C011482* [107].

Tartrate is the primary organic acid in grape berries and an important contributor to the flavor of grape wine [111]. QTLs related to grape tartrate content have been found on chromosomes 1, 2, 4, 6, 7, 8, 9, 13, 16, and 17 [103, 112–114]. *L-idonate 5-dehydrogenase*, encoding a key enzyme in the tartrate synthesis pathway, was reported to be a candidate gene for tartrate content on chromosome 16 [103].

Unusually, for peach fruits, high acidity is a recessive trait [100]. Several QTL mapping studies in peach have demonstrated that the *D* locus, located at the proximal end of LG 5, controls the low-acidity character of peach fruits and co-localizes with major QTLs related to fruit pH, titratable acidity, and organic acid content [115–119]. The SSR marker CPPCT040 and the SNP marker DH875 are tightly linked to the *D* locus [119, 120]. Based on a GWAS conducted in 129 peach accessions, a candidate gene in locus *D*, *ppa006339m* (encoding an auxin-efflux carrier) was proposed to control fruit acidity [17]. In addition, *PpRPH* (*Prupe_5G008400*), a small protein-coding gene, was identified as a strong candidate gene in the *D* locus using comparative transcriptome analysis and transient expression-based functional assays [100]. However, how these two genes regulate organic acid accumulation remains elusive. Interestingly, *PpTST1*, encoding a tonoplast sugar transporter, was also reported to regulate organic acid accumulation, and an SNP (G/T) in the third exon of *PpTST1* was associated with organic acid content [20].

## Quality improvement related to fruit acidity

Fruit flavor is the primary goal of fruit crop breeding programs. In traditional breeding, any selection for flavor phenotypes cannot be carried out until mature fruits are available, which makes breeding time-consuming and laborious. MAS can track traits of interest using associated DNA markers, providing an efficient strategy for earlier selection during the juvenile phase that accelerates traditional breeding [121]. A prerequisite of MAS is the identification of trait-related genetic loci, which is typically achieved by QTL mapping or linkage analysis [122]. During genetic research on the organic acid content of fruit crops, several representative markers were developed based on identified loci or genes. Related markers include the cleaved amplified polymorphic sequences marker CAPS_1455_, corresponding to *Ma1* in apple [40], and an InDel-based CPAS marker for *Sl-ALMT9* in tomato [39]*.* However, there is still a lot of work between the discovery of a QTL, the development of related marker(s), and the practical use of marker(s) in MAS. Additionally, how well a marker predicts a trait should be validated before the application of MAS. Chagné *et al*. [122] chose 25 SNPs on LG 8 and LG 16 related to apple fruit acidity based on the studies of Bai *et al*. [40] and Kumar *et al*. [123], and successfully validated four markers on LG 16 (AHFBAZU, AHHS7CA, AH89247, and AHBAIAO) using the OpenArray^®^ platform. Genomic selection (GS), whereby complex traits are assessed by genome-wide DNA markers, is more effective than MAS. Substantial cost reduction in genotyping makes GS an attractive option for breeding programs; however, the low prediction accuracy of GS is the urgent problem that needs to be addressed. Developing statistical machine-learning models and training population optimization are two promising but challenging areas for improving the prediction accuracy of GS and facilitating wide application of GS in breeding practices [124]. Successful applications of GS to apple fruit quality traits (including titratable acidity) were reported by Kumar *et al*. [125] and Kostick *et al*. [126], which suggested that GS has the potential to assist in early selection of fruit flavor traits and to promote efficient breeding.

For modern agriculture, crop improvement strategies beyond crossbreeding have been proposed, including mutation breeding, genome engineering, and genome editing [127, 128]. Mutation breeding utilizing chemical mutagens or physical irradiation can create random mutations [129]. However, the selection of individuals harboring a desired trait is challenging due to the low frequency of beneficial mutations [128]. Creation of transgenic plants, whereby genes with known functions are transferred into plants using genetic transformation, can directly introduce desired traits into background cultivars to achieve precise quality improvement. Transgenic technology has been successfully adopted for many fruit crops in the laboratory and frequently utilized for gene function research. The creation of transgenic materials with altered organic acid content has laid the foundation for precise quality improvement of fruit crops in the future. Genome editing has opened a new era of fruit crop breeding. The most dramatic example of this is the *de novo* domestication of wild tomato using genome editing [130–132]. In addition, mutagenesis of *CitPH4* and *Sl-ALMT9* by CRISPR/Cas9 has dramatically reduced the citrate content of citrus and the malate content of tomato, respectively [39, 75]. In kiwifruit, *acnac1*-knockout lines showed decreased citrate accumulation [44]. These cases demonstrate that genome editing offers prospects for flavor improvement of fruit crops.

## Conclusions and perspectives

Citrate and malate are predominant organic acids in fruit crops. In terms of malate, reported genetic loci and variations are primarily focused on vacuolar transporters, particularly ALMTs, as well as transcription factors directly regulating these transporters. For citrate, there are a very limited number of genes that have been verified to contain natural variations associated with citrate content (*CitAN1* and *CitPH4* in citrus, *CmPH* in sweet melon, and *ZjACO3* in jujube). Overall, numerous loci for organic acid content have been identified in fruit crops; however, it remains challenging to identify key genes controlling this trait under these loci. The integration of multi-omics and reverse genetics holds great potential as a robust solution to address this issue. Moreover, the identification of key genes regulating fruit acidity in major fruit crops, such as *ALMT*s, facilitates the exploration of homologous genes in other fruit crops. This offers an opportunity to unravel the genetic basis underlying organic acid content in fruit crops lacking high-quality reference genomes or appropriate mapping populations.

The identification of genetic loci and genes regulating organic acids can contribute to molecular breeding and quality improvement of fruit crops. Natural mutations associated with organic acid content can be imitated and recreated by CRISPR/Cas-based genome editing (knockout, knock-in, base editing, etc.) independently of the integration of exogenous DNA fragments into the plant genome, which allows the production of transgene-free cultivars with ideal fruit acidity, thereby achieving precise improvement of fruit crops. Furthermore, with the application of cost-effective and high-throughput genotyping platforms such as SNP chips, identified genetic loci controlling organic acid content could serve MAS in breeding programs and aid in molecular design breeding of fruit crops. However, before this, the universal effectiveness and genetic contribution of these genetic loci need to be further validated in larger populations with different genetic backgrounds.

Both malate and citrate are primary metabolites originating from glycolysis. A potential association between phosphofructokinase (a rate-limiting enzyme in glycolysis) and citrate content was proposed in a QTL mapping study [78]. It is plausible that changes in glycolysis rate could influence the levels of malate and citrate since an excess or deficiency of PEP directly determines the carbon flux into acids. In addition, *MdNADP-ME* was also reported to regulate fructose content [60], indicating the interrelation of acids and sugars. Given that both sugar and acid levels have changed during the domestication process of most fruit crops, there might exist pivotal genes coordinating sugar and acid metabolism which have undergone selection and contributed to the flavor formation of fruits. Deciphering interrelation mechanisms of sugar and acid metabolism would improve the understanding of genetic regulation of organic acid accumulation.

In this review, genetic studies on organic acid content in fruit crops are summarized, providing valuable information for fruit quality improvement. The discovery of citrate tonoplast transporters is expected to facilitate the progress of the genetics of citrate content, and the further exploration of mutual regulation between organic acids and sugars would provide new insights into fruit acidity and flavor formation.

## Supplementary Material

Web_Material_uhae225

## Data Availability

Data sharing is not applicable to this article as no new data were created or analyzed in this study.
