# Supplementary material for: The art of tartness: the genetics of organic acid content in fresh fruits
Source: Hortic Res. 2024 Aug 6;11(10):uhae225. doi: 10.1093/hr/uhae225 (PMC11480666; doi:10.1093/hr/uhae225)
Supplement: Web_Material_uhae225 [file web_material_uhae225.zip › Supplementray meterial.docx]

**Table S1 Genetic loci for organic acid content in tomato identified by GWAS**

| Locus | Chr | Trait | Candidate gene(s) | Gene function | Gene variation & Marker | Reference |
| --- | --- | --- | --- | --- | --- | --- |
| *TFM6* | 6 | Malate | *Sl-ALMT9***^†^**  (*Solyc06g072910*, *Solyc06g072920*) | Malate transporter | A 3-bp InDel in the binging site of WRKY42  ALMT9^indel_3^ CAPS marker | [1,38,84–87] |
| *TFM6* | 6 | Citrate | *Sl-ALMT9*  (*Solyc06g072920*) | – | – | [1,85] |
| *TFM6* | 6 | Malate | *Solyc06g072930.2* | – | – | [86] |
| – | 6 | Malate | *Solyc06g072840* | Hydrogen peroxide-induced protein 1,  might be involved in the TCA cycle | – | [88] |
| *TFM1.1* | 1 | Malate | *Solyc01g010350* | – | – | [87] |
| *TFM1.2* | 1 | Malate | *Solyc01g095910* | – | – | [87] |
| *TFM4.1* | 4 | Malate | *Solyc04g008540* | – | – | [87] |
| *TFM4.2* | 4 | Malate | *Solyc04g008540* | – | – | [87] |
| *TFM6.2* | 6 | Malate | *Solyc06g072440* | – | – | [87] |
| *TFM6.3* | 6 | Malate | *Solyc06g072340* | – | – | [87] |
| *TFM6.4* | 6 | Malate | *Solyc06g083330* | – | – | [87] |
| *TFM6.5* | 6 | Malate | *Solyc06g076360* | – | – | [87] |
| *TFM7.1* | 7 | Malate | *Solyc07g063870* | – | – | [87] |
| *TFM8.1* | 8 | Malate | *Solyc08g067300* | – | – | [87] |
| *TFM8.2* | 8 | Malate | *Solyc08g067210* | – | – | [87] |
| *TFM9.1* | 9 | Malate | *Solyc09g065900* | – | – | [87] |
| *TFM9.2* | 9 | Malate | *Solyc09g072520* | – | – | [87] |
| *TFM12.1* | 12 | Malate | *Solyc12g098630* | – | – | [87] |
| – | 1 | Malate | *Solyc01g008550* | Cinnamoyl CoA reductase-like protein | – | [1] |
| – | 9 | Malate | *Solyc09g098590* | Sucrose synthase | – | [1] |
| – | 11 | Malate | *Solyc11g072700* | Glycosyltransferase-like protein | – | [1] |
| – | 12 | Malate | *Solyc12g008430* | Malic enzyme | – | [1] |
| – | 1 | Citrate | *Solyc01g007090* | ALMT | – | [1] |
| – | 2 | Citrate & malate | *Solyc02g084820* | Glycosyl transferase group 1 | – | [1] |
| – | 3 | Citrate | *Solyc03g083090* | Glycogen synthase | – | [1] |
| – | 7 | Citrate | *Solyc07g055840* | Citrate synthase | – | [1] |
| qTF2.1 | 2 | Citrate | *Solyc02T000402.1* | – | – | [89] |
| qTF2.2 | 2 | Citrate | *Solyc02T000461.1* | – | – | [89] |
| qTF2.3 | 2 | Citrate | *Solyc02T000662.1* | – | – | [89] |
| qTF2.4 | 2 | Citrate | *Solyc02T000684.1* | – | – | [89] |
| qTF3 | 3 | Citrate | *Solyc03T002685.1* | – | – | [89] |
| qTF6.1 | 6 | Citrate | *Solyc06T002476.1* | – | – | [89] |
| qTF6.2 | 6 | Citrate | *Solyc06T002703.1* | – | – | [89] |
| qTF7 | 7 | Citrate | *Solyc07T001849.1* | – | – | [89] |
| qTF9 | 9 | Citrate | *Solyc07T001849.1* | – | – | [89] |
| qTF10.1 | 10 | Citrate | *Solyc10T001673.1* | – | – | [89] |
| qTF10.2 | 10 | Citrate | *Solyc10T002661.1* | – | – | [89] |

**^†^**A Candidate gene which was confirme

Table S2 QTLs for organic acid content in tomato identified by QTL mapping

| Trait | QTL | Chr | Position (Mb) | Population | Reference |
| --- | --- | --- | --- | --- | --- |
| Malic acid | *ma1.1* | 1 | 26.6 | BIL*-S. lyco.*/*S. pimp.* | [92] |
| Malic acid | *ma1.2* | 1 | 76.6 | BIL*-S. lyco.*/*S. pimp.* | [92] |
| Malic acid | *ma1.2* | 1 | – | BC4*-S. lyco./S. peru.* | [93] |
| Malic acid | *ma1.2* | 1 | – | IL*-S. lyco./S. penn.* | [94] |
| Malic acid | *ma1.3* | 1 | 82.6 | BIL*-S. lyco.*/*S. pimp.* | [92] |
| Malic acid | *ma1.3* | 1 | – | BC3*-S. lyco./S. parv.* | [93] |
| Malic acid | *ma1.3* | 1 | – | BC3*-S. lyco./S. neor.* | [93] |
| Malic acid | *ma1.3* | 1 | – | IL*-S. lyco./S. penn.* | [98] |
| Malic acid | *ma2.1* | 2 | – | BC3*-S. lyco./S. hirs.* | [93] |
| Malic acid | *ma2.1* | 2 | – | BC4*-S. lyco./S. peru.* | [93] |
| Malic acid | *ma3.1* | 3 | 38 | BIL*-S. lyco.*/*S. pimp.* | [92] |
| Malic acid | *ma3.2* | 3 | 44 | BIL-*S. lyco.*/*S. pimp.* | [92] |
| Malic acid | *ma3.3* | 3 | 48 | BIL*-S. lyco.*/*S. pimp.* | [92] |
| Malic acid | *ma-4G* | 4 | – | IL*-S. lyco./S. penn.* | [95] |
| Malic acid | *ma-4I* | 4 | – | IL*-S. lyco./S. penn.* | [95] |
| Malic acid | *ma5.1* | 5 | – | BC3*-S. lyco./S. hirs.* | [93] |
| Malic acid | *ma5.2* | 5 | – | BC2*-S. lyco./S. pimp.* | [93] |
| Malic acid | *ma6.1* | 6 | 38.8 | BIL*-S. lyco.*/*S. pimp.* | [92] |
| Malic acid | *ma7.1* | 7 | 0.2 | BIL*-S. lyco.*/*S. pimp.* | [92] |
| Malic acid | *ma7.1* | 7 | – | IL*-S. lyco./S. penn.* | [98] |
| Malic acid | *ma7.2* | 7 | 16.2 | BIL*-S. lyco.*/*S. pimp.* | [92] |
| Malic acid | *ma-8E* | 8 | – | IL*-S. lyco./S. penn.* | [95] |
| Malic acid | *ma9.1* | 9 | 2.7 | BIL*-S. lyco.*/*S. pimp.* | [92] |
| Malic acid | *ma11.1* | 11 | 0.3 | BIL*-S. lyco.*/*S. pimp.* | [92] |
| Malic acid | *MA11* | 11 | – | RIL*-S. lyco./S. pimp.* | [96] |
| Malic acid | *ma-12D* | 12 | – | IL*-S. lyco./S. penn.* | [95] |
| Citric acid | *cca1.1* | 1 | 26.6 | BIL*-S. lyco.*/*S. pimp.* | [92] |
| Citric acid | *cca1.2* | 1 | 72.6 | BIL*-S. lyco.*/*S. pimp.* | [92] |
| Citric acid | *cca2.1* | 2 | 18.2 | BIL*-S. lyco.*/*S. pimp.* | [92] |
| Citric acid | *cca2.1* | 2 | – | IL*-S. lyco./S. penn.* | [98] |
| Citric acid | *cca3.1* | 3 | 16 | BIL*-S. lyco.*/*S. pimp.* | [92] |
| Citric acid | *cca3.2* | 3 | 44 | BIL*-S. lyco.*/*S. pimp.* | [92] |
| Citric acid | *cca3.3* | 3 | 54 | BIL*-S. lyco.*/*S. pimp.* | [92] |
| Citric acid | *cca3.4* | 3 | 56 | BIL*-S. lyco.*/*S. pimp.* | [92] |
| Citric acid | *cca4.1* | 4 | 14.5 | BIL*-S. lyco.*/*S. pimp.* | [92] |
| Citric acid | *ca*-*5E* | 5 | – | IL*-S. lyco.*/*S.penn.* | [95] |
| Citric acid | *ca*-*5E* | 5 | – | IL*-S. lyco.*/*S.penn.* | [97] |
| Citric acid | *cca6.1* | 6 | 4.8 | BIL*-S. lyco.*/*S. pimp.* | [92] |
| Citric acid | *cca6.2* | 6 | 28.8 | BIL*-S. lyco.*/*S. pimp.* | [92] |
| Citric acid | *cca6.3* | 6 | 36.8 | BIL*-S. lyco.*/*S. pimp.* | [92] |
| Citric acid | *cca6.3* | 6 | – | RIL*-S. lyco./S. pimp.* | [96] |
| Citric acid | *cca6.4* | 6 | 38.8 | BIL*-S. lyco.*/*S. pimp.* | [92] |
| Citric acid | *cca7.1* | 7 | 0.2 | BIL*-S. lyco.*/*S. pimp.* | [92] |
| Citric acid | *cca7.1* | 7 | – | IL*-S. lyco./S. penn.* | [95] |
| Citric acid | *cca8.1* | 8 | 0 | BIL*-S. lyco.*/*S. pimp.* | [92] |
| Citric acid | *cca8.1* | 8 | – | IL*-S. lyco./S. penn.* | [95] |
| Citric acid | *cca9.1* | 9 | 50.7 | BIL*-S. lyco.*/*S. pimp.* | [92] |
| Citric acid | *cca9.2* | 9 | 70.7 | BIL*-S. lyco.*/*S. pimp.* | [92] |
| Citric acid | *ca*-*9F* | 9 | – | IL*-S. lyco.*/*S.penn.* | [95] |
| Citric acid | *ca*-*9F* | 9 | – | IL*-S. lyco.*/*S.penn.* | [97] |
| Citric acid | *ca*-10B | 10 | – | IL*-S. lyco.*/*S.penn.* | [95] |
| Citric acid | *ca*-10B | 10 | – | IL*-S. lyco.*/*S.penn.* | [97] |
| Citric acid | *CA11.1* | 11 | – | RIL*-S. lyco./S. pimp.* | [96] |
| Citric acid | *CA11.2* | 11 | – | RIL*-S. lyco./S. pimp.* | [96] |

BC, Backcross line; BIL, Backcross inbred lines; IL, Introgression lines; RIL, Recombinant inbred lines; *S. hirs.*, *Solanum* *hirsutum* *S. lyco.*, *Solanum lycopersicum*; *S. neor.*, *Solanum neorickii*; *S. parv.*, *Solanum parviflorum*; *S. penn.*, *Solanum pennellii*; *S. peru.*, *Solanum peruvianum*; *S. pimp*., *Solanum pimpinellifolium*
